# Embryo-Protective Effects of Cerium Oxide Nanoparticles against Gestational Diabetes in Mice

**Published:** 2018

**Authors:** Zeinab Vafaei-Pour, Mohammad Shokrzadeh, Monireh Jahani, Fatemeh Shaki

**Affiliations:** a *Pharmaceutical Research Center, Faculty of Pharmacy, Mazandaran University of Medical Sciences, Sari, Iran. *; b *Department of Toxicology and Pharmacology, Faculty of Pharmacy, Mazandaran University of Medical Sciences, Sari, Iran.*

**Keywords:** Diabetes, pregnancy, Teratogenicity, Nanoceria, Oxidative Stress

## Abstract

Gestational diabetes is deﬁned as carbohydrate intolerance with onset or ﬁrst recognition during pregnancy. Diabetes during pregnancy increases the incidences of congenital anomalies, in a mother and her embryo. Oxidative stress has been implicated to be responsible in diabetic embryopathy. In this study, we used nanoceria as an antioxidant for amelioration of diabetic embryopathy in diabetic mice. The female mice were divided into 5 groups (6 mice per group). Diabetes was induced by a single dose of streptozotocin (60 mg/kg IP) that dissolved in citrate buffer (pH = 4.6). Blood glucose was checked in 0,5,10, and 15 days of pregnancy. The diabetic state was conﬁrmed when the blood glucose concentration exceeded 200 mg/dL. On the day 16 of pregnancy, all animals were anesthetized with ether and embryos were excised; then oxidative stress, pathological parameters, number of implantations, miscarriage, and live embryo were assayed. Histological study showed that diabetes induced abortion; decrease in weight of mothers, embryo, and the number of embryos were observed. In diabetic mice, significant increase in lipid peroxidation (LPO), ROS formation, and protein carbonyl content were observed. Glutathione (GSH) concentration is found to be decreased in embryo tissue in diabetic mice. Nanoceria treatment significantly inhibited embryonic oxidative stress and also pathologic changes in diabetic mice. Our research showed that diabetes act as a teratogen agent for fetal development and nanoceria abrogated diabetes induced embryopathy via its antioxidant effects. So, early detection of diabetes in pregnancy and antioxidant administration can attenuate these complications.

## Introduction

Gestational diabetes is described as carbohydrate intolerance that results in hyperglycemia, starting or being detected for the first time, during pregnancy (1). Gestational diabetes increases the incidence of complications for both mother and embryo during pregnancy, childbirth, and beyond (2-3).Recent evidence suggested that early detection and management of gestational diabetes could improve outcomes for both mother and child (2-3).

The prevalence of gestational diabetes is most often reported as 2–6% of pregnancies. American Diabetes Association (ADA) guidelines stated that diabetes affects 7% of all pregnancies (4). In fact pregnancy is physiologically ‘diabetogenic’ because of the decrease in tissue sensitivity to insulin and the increase in special antagonists of the insulin, for instance, human placental lactogen (5). Congenital malformations are more seen in infants of diabetic children than women of non-diabetic women (6).

Emerging data suggest that early detection and exact metabolic control of diabetics pregnant women should decline the repetition and intensity of short and long-range complications in the offspring of the diabetic mother (6).The most reported complication of gestational diabetes in neonatal is including congenital anomalies, premature birth, macrosomia, neonatal hyperglycaemia, and infant death (1, 5). Advances during recent years indicate that various teratogen agents can act via increase of oxidative stress in developing embryo and suppression of antioxidant defense system. Also, severe embryonic damage may happen if exposure with these teratogens induced oxidative stress occurred in the early stages of angiogenesis (5, 7-8).

Diabetes conditions and subsequently high glucose concentration induces the production of oxygen and hydroxyl free radicals customarily (9). It seems that hyperglycemia directly increases oxidative stress in embryo tissue and inhibition of oxidative stress could be helpful for improvement of the manifestations associated with diabetes (10). Recent investigations indicated that under oxidative stress conditions (as an allostatic overload), a variety of factors such as overproduction of reactive oxygen/nitrogen species (ROS/RNS) by mitochondria and lipid peroxidation, DNA, and RNA damage are underlying mechanisms linking toxicity to immune-inflammatory responses and oxidative stress (11-12).

Oxidative stress is defined as an imbalance between the production of reactive oxygen species and antioxidant defense capacity of the body which is closely associated with aging and a number of diseases, including cancer, cardiovascular diseases, and diabetes omplications (13). 

There are various studies that reported both types of diabetes can increase oxidative stress in blood and treatment with antioxidants such as vitamin E and flavonoids alleviated oxidative stress and its complication (14-17). Cerium is a rare earth metal of the periodic table that belongs to the lanthanide series. When it combines with oxygen in a nanoparticle formulation, cerium oxide adopts a ﬂuorite crystalline structure that has profound antioxidant properties (18-19).The ability of nanoceria to reversibly bind oxygen and change between Ce^+4^ and Ce^+3^ states under oxidizing and reducing conditions has a significant role in scavenging different types of reactive oxygen (20). The ability of nanoceria to shift between oxidation states is comparable with other biological antioxidant (21-23). Nanoceria has been shown to give protection against ROS and radiation damage (24). Also, cerium oxide (CeO_2_) nanoparticles, has shown a potential new remedy for oxidative disorders that overcomes many of the deﬁciencies of previous therapies for ischemic brain injury (22, 25-26). A constant and collaborative effort has represented the sufficiency of nanoceria to support against cellular damage caused by several radicals in different tissues and organ systems as well as biomedical applications. Also, we focused on protetive effect of nanoceria from teratogenic effect on embryo of diabetic mice.

## Experimental

*Materials*

Stereptozotocine (stz) was purchased from Sigma Chemical Co. ( USA),sodium citrate, Cerium oxide nanoparticles, Comassie blue, ethylenediamine tetra acetic acid (EDTA), 5,5dithiobis-2-nitrobenzoic acid (DTNB), glutation (GSH), Tris-Hcl , 4, 2 hydroxyethyl -1-piperazineethanesulfonic acid (HEPES), Morpholinopropansulfonic acid (MOPS), ethyleneglycol- bis (2-aminoethylether) -N,N,Ń,Ń- tetraacetic acid (EGTA), KCL, Mgcl_2_, KH_2_PO_4_, Succinate, HCL, NaoH, ethanol, ethylacetate, 2´7´ - dicholorofluoresceindiacetate (DCFH-DA), n-butanol, HCL, thiobarbutiric acid (TBA), phosphoric Acid, Trichloroacetic acid **)**TCA),Guanine hydrochloride, 2,4 dinitrophenylhydrazine (DNPH).

*Methods*

*Animal treatment*

Female Swiss albino mice were provided from Laboratory Animals Research Center, Mazandaran University of Medical Sciences, Sari, Iran**.** The animals were housed in an air-conditioned room with controlled temperature of 22 ± 2 °C and maintained on a 12:12 h light cycle with free access to food and water. All experimental procedures were conducted according to the ethical standard and protocols approved by the Committee of Animal Experimentation of Mazandaran University of Medical Sciences, sari, Iran.

**Table 1:** Body weight of pregnant mice in 0,5, 10,15th days of pregnancy.

**Animals group**	**Body Weight (g)** **Mean ± SD**				
	**Day0**	**Day5**	**Day10**	**Day15**	**Weight increase**
Control	29±2.32	34±2.72	44±3.52	54±4.32	25
Diabetic	30±2.4	33±2.64	38±3.04	43±3.44	13*
Nanoceria	30±2.08	36±2.73	45±3.65	53±4.16	23
Diabetic+Nanoceria	31±2.48	36±2.88	41±3.28	49±3.92	18$
Diabetic+vit E	29±2.17	32±2.54	40±3.19	48±4.08	19$

Values are expressed as mean ± SD for six mice in each group,

$ *P *< 0.05vs Diabetic &

* *p *< 0.05 vs control.

**Table 2:** Effects of nanoceria on Body weight (g) of embryos on day of caesarean section

**Mean birth weight**	**Animals group**
0.28 ± 0.03	Control
*0.18 ± 0.1	Diabetic
0.24 ± 0.08	Nanoceria
0.26 ± 0.11^$^	Diabetic+Nanoceria
0.25 ± 0.07^$^	Diabetic+vit E

Values are expressed as mean±SD for, six mice in each group,

* *P *< 0.05 vs control.

**Table 3:** Blood glucose (mg/dL) of mice, in 0,5,10, 15^th^ days of pregnancy

**Animals group**	**Blood glucose (mg/dL)** **mean ±SD**			
	**Day0** **Pregnancy**	**Day5** **Pregnancy**	**Day10** **Pregnancy**	**Day15** **Pregnancy**
Control	97 ± 13.58	97 ± 15.12	100 ± 16.8	103 ±18.2
Diabetic	220 ± 30.8*	287±40.18*	339±47.46*	400 ± 65*
Nanoceria	96 ± 13.22	99 ± 18.43	102±17.58	106 ± 21.37
Diabetic+Nanoceria	212 ± 29.68	270 ± 37.8	323 ± 43.54	372 ± 57.4
Diabetic+vit E	208 ± 27.6	268 ± 35.92		

Values are expressed as mean ± SD for six mice in each group,

* *P *< 0.05 vs control.

**Table 4:** Effects of *in-vivo* administration of Nanoceria and vit E on complication of diabetes in control and diabetic embryos

**Groups**	**number of whole fetuses**		**number of abortion**	
Control		12		0	
Diabetic		6		3	
Nanoceria		10		0	
Diabetic+Nanoceria		9		1	
Diabetic+vit E		7		0	

Values are expressed as mean, for six mice in each group.

**Figure 1:**
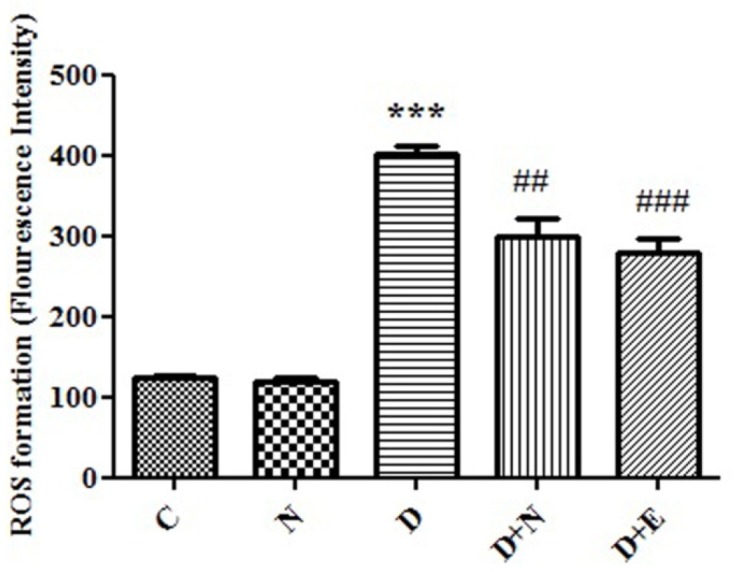
Effect of Nanoceria on ROS formation in embryo tissue. ROS formation was determined in C(Control mice), N( Mice that received Nanoceria for 16 days) , D (Diabetic mice), D+N ( Diabetic mice that received Nanoceria for 16 days), D + E (Diabetic mice that received vit E for 16 days) using DCFH-DA as described in Materials and methods. Values represented as mean ± SD (n = 6). ***P < 0.001 compared with control mice, ^##^*P *< 0.01, ^###^*P *< 0.001 compared with diabetic mice.

**Figure 2:**
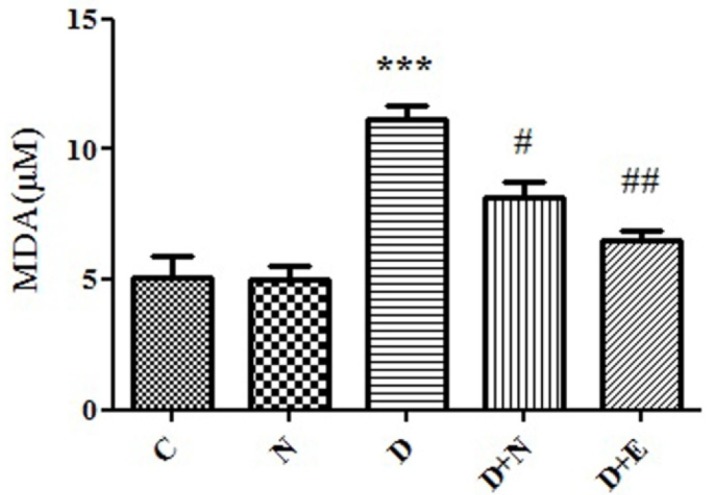
Effect of Nanoceria on lipid peroxidation in embryo tissue**.** MDA level was measured in C(Control mice), N (Mice that received Nanoceria for 16 days), D(Diabetic mice) , D+N ( Diabetic mice that received Nanoceria for 16 days), D+E (Diabetic mice that received vit E for 16 days) using TBA reagent that described in Materials and methods. Values represented as mean ± SD (n = 6). ***P < 0.001 compared with control mice, ^#^*P *< 0.05, ^##^*P *< 0.01 compared with diabetic mice.

**Figure 3:**
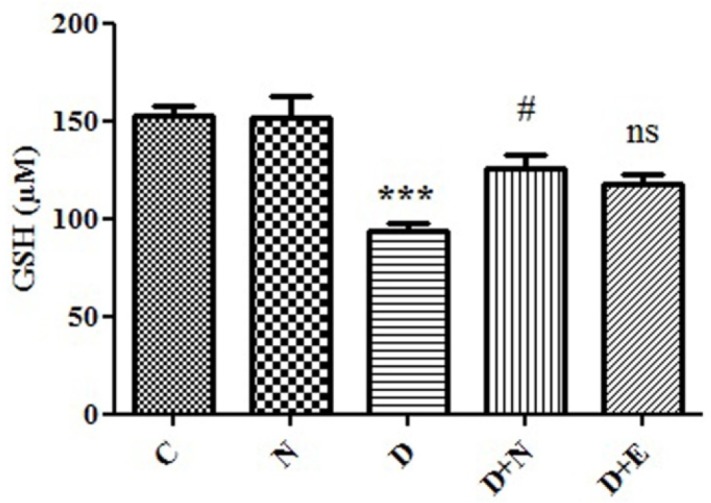
Effect of Nanoceria on GSH concentration in embryo tissue. GSH concentration was measured in C (Control mice), N (Mice that received Nanoceria for 16 days), D(Diabetic mice), D + N (Diabetic mice that received Nanoceria for 16 days), D+E (Diabetic mice that received vit E for 16 days) using DTNB that described in Materials and methods. Values represented as mean ± SD (n = 6). ***P < 0.001 compared with control mice, ^#^*P* < 0.05 compared with diabetic mice.

**Figure 4:**
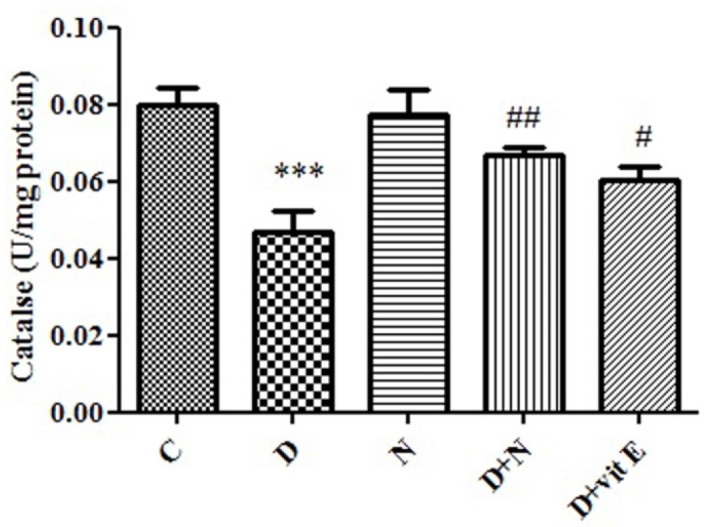
Effect of Nanoceria on Catalase activity in embryo tissue**.** Catalase activity was measured in C (Control mice), N (Mice that received Nanoceria for 16 days), D (Diabetic mice), D + N (Diabetic mice that received Nanoceria for 16 days), D + E (Diabetic mice that received vit E for 16 days). Values represented as mean ± SD (n = 6). ***P < 0.001 compared with control mice, ^#^*P* < 0.05, ^##^*P* < 0.01 compared with diabetic mice.

**Figure 5:**
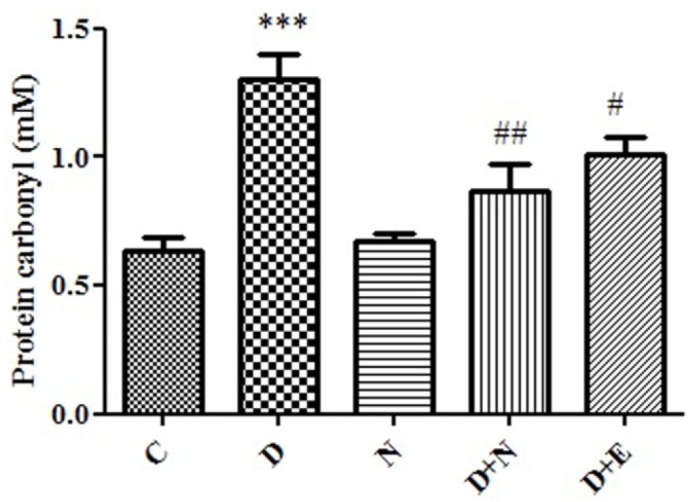
Effect of Nanoceria on protein carbonyl level in embryo tissue**. **protein carbonyl was measured in C (Control mice), N (Mice that received Nanoceria for 16 days), D (Diabetic mice), D + N (Diabetic mice that received Nanoceria for 16 days ), D + E (Diabetic mice that received Vit E for 16 days). Values represented as mean ± SD (n = 6). ***P < 0.001 compared with control mice, ^#^*P *< 0.05, ^##^*P *< 0.01 compared with diabetic mice.

**Figure 6:**
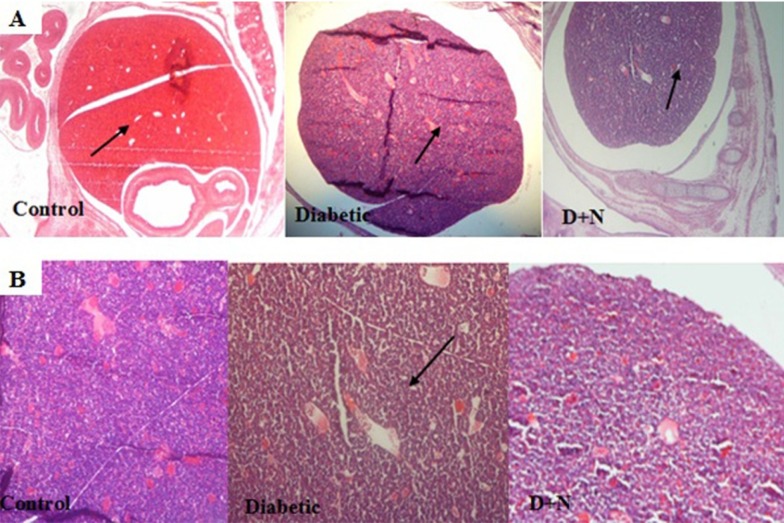
Effects of Nanoceria on diabetic induced pathological changes in liver of mice embryo

Pathological changes in mice embryo of control, diabetic mice and D + N (Diabetic mice that received Nanoceria for 16 days, STZ + Nanoceria group) were examined with H&E staining (200 x). A) Agglomerate in liver tissue in diabetic mice compared with control group, B) necrotic area was shown in the Liver tissue in diabetic group.

**Figure 7:**
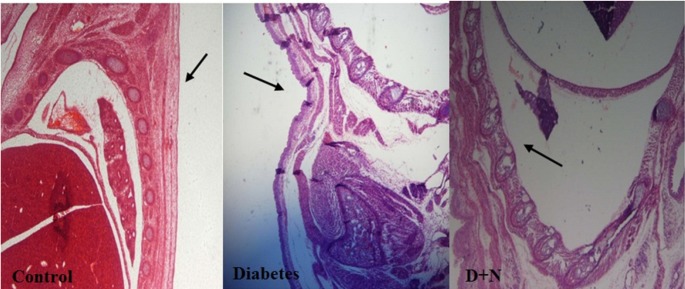
The spine has been found niche in the diabetic group compared with control and D + N (Diabetic mice that received Nanoceria) (200 x).

**Figure 8:**
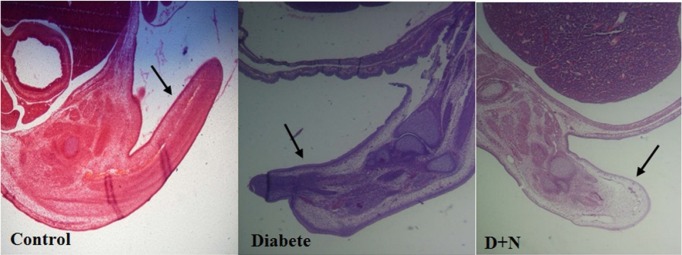
The tail has been found necrotic in the diabetic group compared with control and D + N (Diabetic mice that received Nanoceria) (200 x).

**Figure 9:**
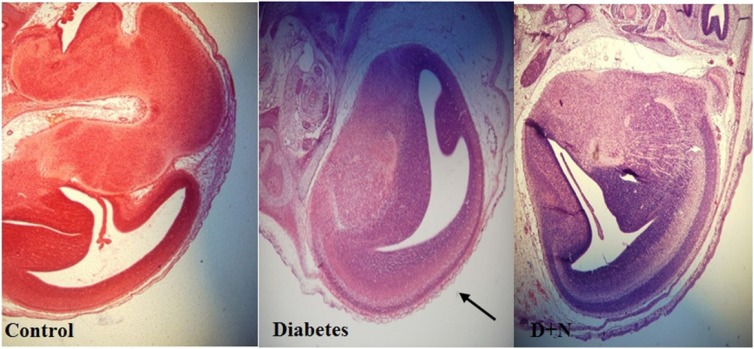
Comparison of the cerebral cortex in embryo of control, diabetic and D + N (Diabetic mice that received Nanoceria) group (200 x).

**Figure 10:**
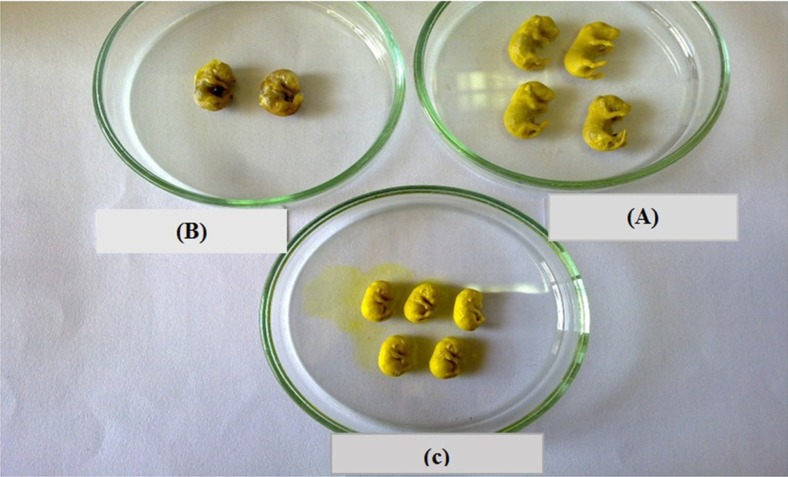
Corruption has become appeared in diabetic group (B), while the condition was the same for all categories (control (A) and Diabetes + Nanoceria (C).

*Experimental design*

The animals were divided into 5 groups, with 6 mice in each group: non-diabetic control mice, mice treated with Nanoceria, diabetic mice, diabetic mice treated with nanoceria (60 mg/kg) for 16 days, diabetic mice treated with vit E (100mg/kg) for 16 days. Diabetes in Female Swiss albino mice was induced by a single dose of intraperitoneal injection of streptozotocin (60 mg/kg) dissolved in citrate buffer (pH = 4.6) (27). One day after STZ administration, the blood was taken from the lateral veins of the tail and blood glucose was measured by a glucometer using the glucose oxidase method. The mice whose blood glucose values were above 200 mg/dL were accepted as diabetic, then all animals were anesthetized and embryos were excised on ice, then some of the embryos was homogenized in phosphate buffered saline, then cen­trifuged at 800xg for 10 min at 4 ℃. The supernatant was collected and oxidative stress markers were assayed.

*Determination of reactive oxygen species (ROS)*

To determine the amount of embryo tissue ROS generation, dichlorofluorescin-diacetate (DCFH-DA) was used as an indicator. Briefly, 2mL of embryo tissue hemoganate (1mg protein/mL) then loaded with DCFH by incubating with this buffer for 15 min at 37 °C. Then, it was monitored at 480 nm (excitation) and at 520 nm (emission) by Shimadzu RF5000U fluorescence spectrophotometer (28).

*Measurement of Lipid peroxidation (LPO):*

The content of MDA was determined by using the method of Zhang *et al* 2008 (29).Also, 0.25 mL phosphoric acid (0.05 M) was added to 0.2 mL of embryo tissue homogenate with the addition of 0.3 mL 0.2% TBA. All the samples were placed in a boiling water bath for 30 min. At the end, the tubes were shifted to an ice-bath and 0.4 mL n-butanol was added to each tube. Then, they were centrifuged at 3500 rpm for 10 min. The amount of MDA formed in each of the samples was assessed through measuring the absorbance of the supernatant at 532 nm with an ELISA reader (Tecan, Rainbow Thermo, Austria). Tetramethoxypropane (TEP) was used as standard and MDA content was expressed as nmol/mg protein.

*Measurement of glutathione content*:

Glutathione (GSH) content was determined by DTNB as an indicator and spectrophotometer. Brifely, 0.1 mL of embryo tissue was added into 0.1 mol/l of phosphate buffers and 0.04% DTNB in a total volume of 3.0 mL (pH 7.4). Then, developed yellow color was read at 412 nm on a spectrophotometer (UV-1601 PC, Shimadzu, Japan). GSH content was expressed as μg/mg protein (30).

*Measurement of Protein Carbonyle*:

It was determined by spectrophotometric method with minor modification (31). Briefly, 200μL of the hemogenate embryo tissue sample was extracted in 500 μL of 20% (w/v) TCA. Then, the Samples placed at 4 °C for 15 min. The precipitates were treated with 500 μL of 0.2% DNPH and 500 μL of 2M NaOH for the control group, and the samples were incubated at room temperature for 1 h with vortex in at 5-min intervals. Then, the proteins are precipitated by adding 55 μL of 20% TCA. The micro-tubes are centrifuged and washed three times with 1000 μL of the ethanol-ethyl acetate mixture. The micro-tubes are dissolved in 200 μL of 6 M guanidine hydrochloride. The carbonyl content is determined by reading the absorbance at 365 nm wavelength.

*Measurement of Catalase activity*:

Catalase activity was assayed by measuring the absorbance decrease at 240 nm in a reaction medium containing H_2_O_2_ (10 mM), sodium phosphate buffer (50 mM, pH: 7.0). One unit of the enzyme is defined as 1 mol H_2_O_2_ as substrate consumed/min, and the specific activity is reported as units/mg protein (32).

*Measurement of protein concentration*: 

Protein content was determined in embryo tissue with Bradford method (33). Bovine serum Albumin (BSA) was used as standard, homogenate samples mixed with coomassie blue and after 10 min, absorbance was determined at 595nm by spectrophotometer.

*Pathological Investigation*

Firstly Animals were anesthetized by ether, then the embryos were drived out by caesarean section from the control and tested mice, and washed with physiologic serum and fixed in bouin for 18 h, then dehydrated in a graded series of ethanols and also we used toluene for extracting alcohol, after we used paraffin in oven for tissue and rapidly, tissues saturated by paraffin and after 4 h the samples fixed on microtome and sections with thickness of 10 micrometer were obtained. Then, the sections were transferred on the slides. Finally, for assessment with light microscope were stained with hematoxylin and eosin (34).

*Statistical analysis*

Results are presented as mean ± SD. All statistical analyses were performed using the SPSS software, version 20. Assays were performed in triplicate and the mean was used for statistical analysis. Statistical significance was determined using the one-way )ANOVA( test, followed by the post-hoc Tukey test. Statistical significance was set at (*P* < 0.05).

## Results

*Effects of Nanoceria on Body weight (g) in diabetic pregnant mice:* Results obtained from *t*-test analysis revealed that there was a significant decrease in weight in diabetic mice when compared with control group (*P *< 0.05; Table.1). Also, diabetic mice that were treated with nanoceria for 16 days were shown significant weight gain than what was seen in diabetics group (Table 1).

*Effects of Nanoceria on Body weight (g) of embryos*

As shown in Table 2, treatment of all embryos of diabetic mice resulted in significant reduction of weight compared to the control group, but when nanoceria administrated during pregnancy in diabetic mice, significant increase in embryos weight was observed in comparison to diabetic group (Table 2).

*Effects of Nanoceria on Blood glucose in diabetic pregnant mice*

The one-way (ANOVA) analysis indicated the significant increase in glucose level (*P* < 0.001). There was no significant change in blood glucose level between diabetic mice group and nanoceria treated diabetic mice group (*P *< 0.05; Table 3).

*Effects of Nanoceria on complication in embryos*

As shown in Table 4, the number of embryos caused a significant increase in diabetic mice compared with control group. Besides, the rate of anomalies and spontaneous abortion increased in diabetic mice compared to control group (*P* < 0.0001).

*Effects of nanoceria on ROS formation in embryo*

ROS formation, as indicator of oxidative stress was significantly increased in diabetic mice compared to control groups (*P *< 0.05). Furthermore, nanoceria could significantly decreased ROS generation in diabetic mice. On the other hand, treated with vit E no significant change in ROS formation in embryo tissue compared to diabetic mice groups (Figure1).

*Effects of Nanoceria on Lipid Peroxidation in embryo*

One of the end products of LPO is malondialdehyde (MDA) and elevation of MDA is known as an important marker for oxidative stress. MDA level was increased in embryo significantly (*P *< 0.05) as compared to control group. As indicated, diabetes induced LPO significantly was inhibited by nanoceria treatment respect to vit E treated groups (Figure 2).

*Effects of Nanoceria on GSH concentration in embryo* **:**

Generally imbalance between ROS and antioxidant levels, such as GSH in diabetic mother caused oxidative stress-induced embryo damage. As shown in Figure 3, GSH level in diabetic mice groups decreased to 94 μM in embryo tissue as compared to control group (153μM). Our results showed that GSH level in diabetic mice that received nanoceria for 16 days was significantly increased compared to diabetic mice (*P *< 0.05). However, there were no significant differences in the GSH levels between vit E and diabetic mice treated groups (*P *> 0.05).

*Effects of Nanoceria on Catalase activity in embryo:*

In diabetic group, there was a significant decrease in CAT activity in embryo tissue (*P *< 0.001) compared to control mice. Administration of nanoceria significantly (*P *< 0.05) decreased catalase activity in diabetic mice as compared to control group and vit E had lower effect than Nanoceria (Figure 4).

*Effects of Nanoceria on Protein carbonyl in embryo*: 

Protein Carbonyl is an indicator of protein oxidation in diabetic patients. There was a significant increase in the protein carbonyl levels in diabetic mice groups (*P *< 0.05). Also, there was significant difference between nanoceria treatment groups as well as vit E group with diabetic mice groups (*p *< 0.05). (Figure 5).

*Effects of Nanoceria on Histological examination in embryo*

As shown, histological studies in embryo of STZ-induced diabetic mice showed some morphology changes in embryo. Nanoceria treatment prevented these pathologic changes in embryo that was similar to vit E treatment Figures 6-10.

Pathological changes in mice embryo of control, diabetic mice and D + N (Diabetic mice that received Nanoceria for 16 days, STZ + Nanoceria group) were examined with H&E staining (200 x). A) Agglomerate in liver tissue in diabetic mice compared with control group, B) necrotic area was shown in the Liver tissue in diabetic group.

## Discussion

The current study was conducted to evaluate whether administration of nanoceria particles (60 mg/kg) has protective effects against embryonic oxidative stress biomarkers and also a pathological profile in embryo of STZ-induced diabetic mice. Our findings clearly demonstrated that the administration of nanoceria at a dose of 60 mg/Kg without any significant effect on the serum glucose levels, reverse the elevation of oxidative stress markers to the normal values. Moreover, diabetes-induced malformation in visceral and spinal of embryo, partially restored by nanoceria treatment. Taken together, nanoceria prevented diabetes-induced teratogenicity in mice, and these novel findings provided new insights into using antioxidant for protection against diabetes induced side effects in pregnancy. There is increasing evidence that demonstrated ROS have an important role in the pathology of numerous congenital anomalies such as those produced by gestational diabetes, radiation, or alcohol and cocaine intake. This mechanism of action also was suggested for some teratogenic drugs such as phenytoin and thalidomide (11, 35-36). Recently, much work revealed that oxidative stress plays an important role in the pathogenesis of diabetes complication. Experimental and clinical studies have shown that hyperglycemia via several biochemical pathways, including glucose autoxidation and protein glycation, leads to increase ROS formation (37-38). Also, it has been seemed that developing embryos are very sensitive to high concentration of ROS, especially during the organogenesis stage (11, 39).

On the other hand, increased levels of ROS in mothers with pregestational diabetes mellitus may cause elevation of lipid peroxidation, protein oxidation, DNA and RNA damage in the developing embryo. So, these pathological events lead to more incidences of brain, cardiovascular, skeletal and other anomalies in offspring of diabetic women than offspring of non-diabetic women (5, 10, 40). We observed that increased level of ROS, lipid peroxidation and protein carbonyl and suppression of enzymatic and non-enzymatic antioxidant in embryo of diabetic mice that were in agreement with previous studies. Interestingly, there was a correlation between the rate of anomalies and the degree of oxidative stress in embryo. However, it has been shown that even with the best glycemic control, the rate of complication is still high. Current treatment options do not target oxidative stress and preventing or inhibiting oxidative stress may be helpful as an obvious and promising potential target for treatment of diabetes complication. Cerium oxide is a rare-earth oxide that is found in the lanthanide series of the periodic table (41-42). Nanoceria has emerged as a lucrative material in biomedical science due to its unique ability to switch oxidation states between (III) and (IV) depending upon the environment. The ability to switch between mixed oxidation states of nanoceria is comparable to biological antioxidants. This imparts nanoceria with a very important biological property of radical scavenging which can be tuned based upon the retention of oxygen vacancies (defects) and concentration of Ce^3+^ species in nanoceria (42-43).Numerous studies have demonstrated that cerium oxide nanoparticles possess antioxidant properties as they improve total antioxidant capacity, suppress destructive oxygen free radicals and prevent oxidative stress damage (22, 44-45). There is no evidence about the effect of nanoceria on embryonic biomarkers of oxidative stress until now, and this study is the first one in this regards. Our data showed that nanoceria significantly inhibited the increase in embryonic ROS production, LPO and protein oxidation. Also, embryonic antioxidant capacity significantly increased following the treatment of diabetic mice with 60 mg/Kg nanoceria. It is important to note that the nanoceria had better antioxidant effects in embryo of diabetic pregnant mice rather than vit E. Previous studies showed that using antioxidant vitamins such as vitamin E, vitamin C and folic acid, is not enough and nowadays there is a tendency to use plant flavonoids, or synthetic products that in addition to have a strong antioxidant effect, have also low toxicity. So, one of the most effective ways to reduce congenital abnormality is detecting effective antioxidant with low complications. Nanoceria as an antioxidant like vitamin C and E can reduce oxidative stress and lipid peroxidation and increase glutathione capacity but with more antioxidant power (19, 46). Cerium oxide (CeO_2_) nanoparticles were thought to increase antioxidant power due to their catalytic effect in stimulating superoxide dismutase (SOD) activity and detoxifying free radicals by remaining active in tissues for extended periods through moving spontaneously between the oxidized and reduced state (18, 47-49).In our study we saw decrease in weight of diabetic mice compare with control group and by administrating nanoceria during pregnancy the diabetic group gained more weight. Also, treatment with nanoceria improved histopathological changes in embryo tissue. Interestingly, the administration of 60 mg/kg nanoceria has antioxidant property without any significant effect on glucose level that confirmed the beneficial role of antioxidants in attenuating diabetes embryopathy. 

## Conclusion

Nanoceria via suppression of oxidative stress signaling was helpful in inhibiting gestational diabetes complication in mice. On the basis of promising effects of nanoceria in animal model, it can be considered as a suitable candidate for future studies in diabetic patients.
